# Political leadership for women’s, children’s and adolescents’ health 

**DOI:** 10.2471/BLT.16.174367

**Published:** 2016-05-02

**Authors:** Nila Moeloek, Kesetebirhan Admasu

**Affiliations:** a Ministry of Health Indonesia, Lv. 2 Adhyatma, Building Jl, HR Rasuna, Said Blok X5, Kav. 4-9, Jakarta, Indonesia.; b Ministry of Health Ethiopia, Addis Ababa, Ethiopia.

The sustainable development goals (SDGs) cannot be achieved without committed political leadership. Progress on women’s, children’s and adolescents’ health, requires such commitments to be upheld and translated into concrete action across sectors.^1^ Since 2000, the eight goals of the millennium development goals (MDGs) inspired our governments to improve health and socioeconomic development. Despite unfinished business, this is a strong foundation for the transition to the sustainable development goals (SDGs).^2^

The *Global strategy for women’s, children’s and adolescents’ health (2016–2030)* is a platform to support country-led actions to operationalize the SDGs.^3^ Indonesia and Ethiopia are two countries that made active contributions to the formulation of this global strategy. As ministers of health, we are placing women, children and adolescents at the core of our governments’ national health policy. Our governments have incorporated local and community-level efforts by a range of stakeholders. Citizen leadership has provided us with insight when formulating our national health policies. In 2015, we held national citizens’ hearings with young people to get input into our national plans and the global strategy.

In the transition to implementation, we welcome discussions on the SDGs and the global strategy at this month’s World Health Assembly. We seek to learn from other countries’ successes and challenges and would like to share some of our own.

In Indonesia, between 2000 and 2015 the percentage of Indonesians living in poverty decreased from 19% to 11% and the lives of millions of women, children and adolescents have been improved by increased health budgets and health service coverage.^4^However, keeping in mind the multiple dimensions of health and poverty, challenges remain. More than half of Indonesian women have access to maternal health care and more than two-thirds of babies are born in health facilities. Despite these improvements, fifty percent of all under-five deaths in Indonesia occur in the first month of life and we recognize that newborn health requires greater investment and attention. There are also those who experience difficulty in accessing health services due to challenges beyond health, such as remote geographical locations and lack of transportation and other facilities. ^5^

The Indonesian government is also striving towards better laws for reproductive health to strengthen this component of the health system, improve the quality of care and extend coverage, especially to those living in hard-to-reach places. With the aim of achieving universal health coverage by 2019, the government is implementing a national health insurance system that includes maternal care, childbirth services and newborn care. Eight hundred thousand pregnant women have already used these services in 2016.

Indonesia has more than 65 million adolescents and young people aged 10 to 24 years. With the great number of young people, the demographic dividend for the Indonesian population is very high if these young people can reach their full potential, and have opportunities to contribute to national development. This requires the achievement of social, economic and environmental development goals. The existence of adolescent-friendly health services and a cross-sectoral national strategy for adolescent health, reflect the commitment by the government to improve sexual and reproductive health education^6^ and adolescent health and development overall.

In Ethiopia, since 1990, under-five deaths have declined by over two-thirds and maternal deaths have declined by almost three-quarters.^7^ Under-five mortality reductions were achieved by focusing on essential immunizations, malaria prevention and treatment, vitamin A supplementation, birth spacing and early and exclusive breastfeeding; all combined with socioeconomic improvements. To achieve maternal mortality reduction, the government instituted accelerated midwifery training and scaled up family planning; access to modern contraceptive methods for women doubled within five years.^1^ The government has also instituted reforms to fight corruption and improve the efficiency of the civil services.^8^ However, more needs to be done to tackle neonatal mortality, improve adolescents’ health and improve opportunities for adolescents to participate fully in society. 

Ethiopia is addressing these and other major challenges in the health sector through its health extension programme, which has expanded health service coverage, particularly for the rural poor.^8^ The government has started *Grand Challenges Ethiopia* to introduce proven innovations for maternal, newborn health and early childhood development into its health system.^9^Ethiopian adolescents and young people are engaged through health programmes in schools, universities and youth centres.^10^ The importance of a highly-skilled and well-resourced health workforce is recognized and the necessity for monitoring systems to generate data on which to base health decisions, is understood. The government is working to eliminate child marriage and female genital mutilation.^11^

Now, as we move into the SDG era, we begin by sending a clear and strong message from our governments; “we are committed”. We have accountable leadership and we support global cooperation to drive progress on women’s, children’s and adolescents’ health. In the spirit of the partnership among equals emphasized in *Transforming our world: the 2030 agenda for sustainable development*,^2^ let us, political leaders, continue to be open to new ideas from our national stakeholders and other countries and share our knowledge and experiences. 
